# *Leveling the playing field* – Kommunikationsstrategien und Ungleichheitsnarrative des linken Diskurses im Umfeld der Demokratischen Partei der USA

**DOI:** 10.1007/s41358-021-00275-2

**Published:** 2021-08-04

**Authors:** Natalie Rauscher, Maren Schäfer

**Affiliations:** Heidelberg Center for American Studies (HCA), Ruprecht-Karls-Universität, Heidelberg, Deutschland

**Keywords:** Ökonomische Ungleichheit, Narrative, Framing, Politische Kommunikation, US Vorwahlen, Economic inequality, Narratives, Framing, Political communication, US primaries

## Abstract

In den letzten Jahrzehnten ist in den USA eine größer werdende Einkommens- und Vermögensungleichheit zu beobachten. Zusätzlich hat die Präsidentschaft Donald Trumps die Spaltung im Land weiter vertieft. Vor diesem Hintergrund ist das entscheidende Narrativ der amerikanischen Gesellschaft unter Druck geraten: Der *American Dream*. Diese Erzählung verspricht jedem Chancengleichheit und wirtschaftlichen Aufstieg. Ein Versprechen, dass für viele Amerikaner:innen allerdings nicht mehr der Wirklichkeit entspricht. Vor allem junge Leute fordern deshalb tiefgreifende Veränderungen im politischen System und eine Umkehr der etablierten Politik. Viele dieser Wähler:innen richten sich zunehmend nach links aus und legen vermehrt Wert auf die Diskussion progressiver Themen innerhalb der Demokratischen Partei. Obwohl sich Kandidat:innen des linken Spektrums im Vorwahlkampf 2020 nur bedingt durchsetzen konnten, gehörten progressive politische Schwergewichte wie Bernie Sanders oder Elizabeth Warren lange zu den Favoriten für die Präsidentschaftskandidatur.

Elizabeth Warren scheiterte im Wahlkampf, konnte jedoch bei vielen Themen Schwerpunkte setzen und trieb oftmals die Agenda voran. Deshalb lässt eine Analyse ihrer Kommunikation Rückschlüsse auf den linken Diskurs innerhalb der Demokratischen Partei der USA zu. An ihrem Beispiel zeigt dieses Papier daher, welche Ungleichheitsnarrative im (progressiven) politischen Diskurs der Vereinigten Staaten eine Rolle spielen und wie sie kommuniziert und mit Hilfe von Frames bewertet werden. Eine Korpus-gestützte Frame-Analyse zeigt beispielhaft die Verwendung zweier Frames im politischen Diskurs: Des „*fairness*-Frames“ und des „*wealth*-Frames.“ Diese heben hervor, wie stark der *American Dream* für den Durchschnittsamerikaner unter Druck geraten ist und legen nahe, warum sich ein größerer Teil der amerikanischen Wählerschaft Kandidat:innen zuwenden, die strukturelle Veränderungen des amerikanischen Systems befürworten.

## Abstract (en)

Over the last decades, the U.S. has seen increasing income inequality and a widening wealth gap. Donald Trump’s presidency has deepened the political and cultural divide among Americans even more. As a result of these developments, many Americans have started to question the idea of the *American Dream*. For many, its promises of equal opportunity and upward social mobility no longer hold true. Young Americans, who are particularly affected by these trends, have started to demand big structural change in the American political and economic system. In line with that, they have championed progressive reform ideas and actors in the Democratic Party. While progressive politicians such as Bernie Sanders or Elizabeth Warren did not secure the presidency in 2020, they were among the Democratic Party’s highest-polling contenders for a considerable portion of the campaign.

Although Elizabeth Warren eventually dropped out of the presidential race, she often managed to drive its political agenda. Taking her strategic communication during the 2019/2020 primaries as an example, this paper analyzes which narratives of inequality are utilized in American progressive political discourse, how these are communicated, and which interpretations of economic inequality her strategic framing suggests. A corpus-based analysis of Warren’s framing reveals that two frames are prevalent in her discourse on inequality: the *fairness*-frame and the *wealth*-frame. These highlight to what extent the *American Dream *has proven unattainable to many Americans and indicate why an increasing number of voters support candidates who demand structural change in the US.

## Einleitung

In den letzten Jahrzehnten, und nicht zuletzt seit der Finanzkrise 2008, ist in den USA eine immer größer werdende Einkommens- und Vermögensungleichheit zu beobachten. Infolgedessen fehlen großen Teilen der amerikanischen Bevölkerung zunehmend die Mittel, um ein Mittelschichtsdasein zu finanzieren. Mit dieser Entwicklung geht einher, dass das entscheidende Narrativ der amerikanischen Gesellschaft stark unter Druck gerät: Der *American Dream*. Dieser hat der amerikanischen Bevölkerung seit jeher versprochen, dass ein besseres Leben und sozialer Aufstieg für jeden möglich seien, wenn man nur hart genug dafür arbeite – Chancengleichheit und wirtschaftlicher Aufstieg bilden also den Kern dieser Idee. Auch die oft zitierte Vorstellung, sich ‚vom Tellerwäscher zum Millionär‘ hocharbeiten zu können, ist eine Ausprägung dieses Narrativs.

Narrative sind entscheidend, um menschliches Handeln zu verstehen und zu interpretieren, sei es bei politischen oder ökonomischen Entscheidungen. Im Feld der Ökonomie betont Robert Shiller die Signifikanz von Narrativen, um wirtschaftliches Handeln zu verstehen (Shiller 2018). Narrative sind als populäre Geschichten oder einfach Erklärungen zu verstehen, die durch die Geschichtsschreibung hinweg immer wieder in gleicher oder veränderter Form auftreten können. Narrative enthalten oft Elemente, die sich auf echte Erfahrungen stützen, können aber auch nur auf Gerüchten oder falschen Tatsachen beruhen (Shiller 2017, S. 968). Durch ihre Verbreitung und deren universelle Popularität formen Narrative den öffentlichen und politischen Diskurs zu einem bestimmten Thema aus und treiben ihn voran. Somit können Diskurse und Narrative menschliches Handeln beeinflussen, denn sie sind eine Möglichkeit, die Wirklichkeit zu interpretieren und danach zu handeln. Auch im politischen Diskurs wird diese „Kulturtechnik des Erzählens“ (Yildiz et al. 2018, S. 136) von politischen Handlungsträgern:innen genutzt. Hierbei muss das Augenmerk zudem auf gezielte sprachliche Strategien gelegt werden, die neben parteipolitischen Positionen und der „Kraft der besseren Argumente Legitimitäten“ (Yildiz et al. 2018, S. 136) vermitteln.

Allem Anschein nach können sich aber mit dem Narrativ des *American Dream*, der die Aussicht auf ein besseres Leben verkörpert, immer weniger Bevölkerungsteile in den USA identifizieren. Die amerikanische Bevölkerung verliert zunehmend das Vertrauen in die Fähigkeit der etablierten Politik und der Gesellschaft, ein gutes Leben und ökonomische Sicherheit zu ermöglichen. Fehlende Mobilität zwischen Gesellschaftsschichten und systemische Probleme lassen viele an der Möglichkeit, ihren *American Dream* zu leben, zweifeln. Diese Probleme lassen sich auch anhand von Zahlen belegen: Die absolute Einkommensmobilität in den USA ist seit den 1940ern stark zurückgegangen. Die Mehrheit der amerikanischen Bevölkerung, die in den 40er-Jahren geboren wurde (ca. 90 % der Geburtenkohorte), verdiente am Ende mehr als ihre Elterngeneration; für die Geburtenkohorte der 80er-Jahre ist diese Zahl auf 50 % gefallen (Chetty et al. 2016, S. 2). Die stärksten Rückgänge finden sich in den früheren industriellen Zentren der USA im mittleren Westen, dem sogenannten Rust Belt. Hier haben viele Menschen den Eindruck – und die Zahlen bestätigen dies –, dass es ihnen wirtschaftlich schlechter geht als früheren Generationen (Chetty et al. 2016, S. 4). Diese Entwicklung hat den Glauben an das Narrativ des *American Dream* nachhaltig erschüttert.

In diesem Zuge scheinen immer weniger Menschen den etablierten Akteur:innen zuzutrauen, sie aus dieser Misere herauszuführen. Seit der letzten Wirtschaftskrise und den Jahren der Obama Regierung, die zwar von einem stetigen, aber langsamen Aufschwung geprägt waren, haben sich viele Teile der Bevölkerung zunehmend alternativen Kandidat:innen zugewandt, sowohl am linken als auch am rechten Ende des politischen Spektrums. Zwar sind dafür nicht nur wirtschaftliche Umstände ausschlaggebend, doch ist dies als signifikanter Grund anzusehen, warum sich ein großer Teil der Wählerschaft dafür entscheidet, Kandidat:innen außerhalb des sogenannten Establishments zu wählen. Einige Kandidat:innen, die *big structural change* unterstützen, konnten in den letzten Jahren Wahlerfolge erzielen. Sie wollen zeigen, dass die USA ihrem Versprechen eines besseren Lebens, dem *American Dream*, wieder gerecht werden können, wenn man systemische Probleme revidiert. Am Beispiel von Elizabeth Warrens Wahlkampf im Rennen um das Präsidentenamt im Jahr 2019/2020 wird in diesem Artikel exemplarisch gezeigt, wie Politiker:innen progressive Politikansätze strategisch kommunizieren.

Das Bekämpfen der Ungleichheit in den USA und die Wiederbelebung des *American Dream* sind und bleiben zentrale Anliegen progressiver Politiker:innen wie Warren und des insgesamt erstarkten progressiven Flügels der Demokratischen Partei. Auch, wenn Joe Biden selbst nicht zu diesem Flügel zu rechnen ist, spiegelte seine Präsidentschaftskampagne, die er zusammen mit Kamala Harris führte, den stärker werdenden Einfluss des linken Parteiflügels wider, der inzwischen von keinem in der Demokratischen Partei noch ignoriert werden kann. Obwohl noch nicht klar ist, wie lange der „Burgfrieden“ zwischen moderaten und progressiven Kräften in der Partei nach der Wahl halten wird, der sich vor allem darauf gründete, eine weitere Amtszeit Donald Trumps zu verhindern, ist nicht von der Hand zu weisen, dass Ideen, wie Elizabeth Warren sie vertritt, zunehmend Teil des politischen Mainstreams in der Demokratischen Partei geworden sind. Auch außerhalb der Demokratischen Partei scheinen einige dieser Themen an Einfluss zu gewinnen, denn unabhängig von Siegen oder Niederlagen der zwei politischen Parteien konnten in einigen Staaten progressive Vorhaben wie ein höherer Mindestlohn oder die Dekriminalisierung von Drogenkonsum durch direkte Abstimmungen durchgesetzt werden (Thunert 2021).

Dieses Papier zeigt, welche narrativen Muster Elizabeth Warren im Zusammenhang mit ökonomischer Ungleichheit verwendet und wie diese strategisch kommuniziert werden, um ihrer politischen Agenda Legitimität zu verleihen. Trotz ihres letztlich schlechten Abschneidens im Vorwahlkampf hat Warren mit ihren narrativen Mustern möglicherweise einige entscheidende diskursive Erfolge erzielt. Ihre sprachliche Strategie gibt deshalb nicht nur Auskunft über ihre eigenen Anliegen, sondern lässt auch Rückschlüsse auf die Anliegen und kommunikativen Strategien des progressiven Flügels der Demokratischen Partei zu, der zunehmend *structural change* – also einen fundamentalen Umbau weiter Teile des amerikanischen Wohlfahrtsstaates und eine Adressierung der Probleme, die das amerikanische Wirtschaftssystem hervorruft – fordert. Hierbei bewegt sich Warrens Sprache eng am Narrativ des *American Dream*, dessen Versprechen sie durch ihre Politikvorhaben für die Mehrheit der amerikanischen Bevölkerung wieder erfüllen will.

Um herauszufinden, wie genau Warren das Problem der ökonomischen Ungleichheit, Ausdruck des gebrochenen Versprechens des *American Dream*, in ihrer Kommunikation darstellt, wurde eine Korpus-gestützte Frame-Analyse ihrer Policy Vorschläge^1^ aus dem Präsidentschaftswahlkampf durchgeführt. Diese zeigt, dass Warren die amerikanische Gesellschaft mit Hilfe von Frames bewertet, die ihrer möglichen Wählerschaft ökonomische Ungleichheit als systemisches Problem präsentieren. Warrens Kommunikation hebt hervor, dass die USA ihrem Versprechen von Fairness, Chancengleichheit und Anerkennung harter Arbeit gerecht werden müssen und dafür eine grundlegend andere Politik und die Umverteilung des Vermögens notwendig sind. Dabei entfaltet diese Art der Kommunikation ihre Wirkung vor allem durch aktive Nutzung von Social Media, ohne die ein erfolgreicher Wahlkampf heutzutage unmöglich scheint.

## Der Ungleichheitsdiskurs in den USA heute

In den letzten Jahren wurden in den USA Themen wie die wachsende Ungleichheit hinsichtlich Einkommen und Vermögen, ein höherer Mindestlohn, Zugang zu einer bezahlbaren Krankenversicherung oder die Dekriminalisierung von Drogenkonsum zunehmend diskutiert. Jüngere Generationen wie die Millennials unterstützen verstärkt diese Themen und entsprechende politische Kandidat:innen, meist auf Demokratischer Seite.^2^

Politiker:innen wie Elizabeth Warren, die seit Jahren eine Kritikerin großer Banken und des Einflusses Vermögender in der Politik ist, profitieren von der Verschiebung des Diskurses nach links. Die Themen, die seit langem auf ihrer Agenda stehen, beschäftigen nun auch moderatere Kräfte in der Partei. Dabei ist sie von sich selbst als ‚demokratischen Sozialisten‘ bezeichnenden Politikern wie Bernie Sanders abzugrenzen. Obwohl auch Bernie Sanders nach europäischer Auffassung eher als Sozialdemokrat skandinavischer Färbung einzuschätzen ist, der einen starken Staat und ein deutlich ausgebauteres Wohlfahrtssystem wie etwa in Dänemark auch für die USA fordert, sieht sich Elizabeth Warren im Grunde als eine Verfechterin des kapitalistischen Systems. Sie möchte gleiche Chancen für alle innerhalb des Marktsystems der USA wiederherstellen. Zudem wollte sich Warren im Präsidentschaftswahlkampf 2019/2020 als eine Alternative zwischen dem linken Pol Bernie Sanders und dem moderaten Flügel der Demokratischen Partei, vertreten durch den letzlich erfolgreichen Joe Biden, darstellen. In Abgrenzung zu Bernie Sanders, der stehts für ein ‚grassroots movement‘ plädiert^3^, war Warren sicherlich die Alternative der gut gebildeten Bürgerschicht, die sich links vom moderaten Flügel der Demokraten verortet. Letzendlich muss man allerdings sagen, dass Warren mit dieser Strategie im Wahlkampf nicht erfrolgreich war (Friedersdorf 2020).

Warren sagt selbst von sich, sie sei „a capitalist to my bones“ (Economist 2019) und vertritt die Auffassung: „I love what markets can do … They are what make us rich, they are what create opportunity“ (Economist 2019). Warren sieht immense Chancen im kapitalistischen System, doch nur, wenn die Märkte fair agieren, was sie ihrer Auffassung nach nicht mehr tun. Dabei ist ihre Kommunikation geprägt von dem Narrativ des *American Dream*. Sie will Chancengleichheit (*equality of opportunity*) und Fairness für alle zurückbringen. *Leveling the playing field* ist das Ziel und Lösungen für ein *rigged system*, das die Mittelschicht benachteiligt, müssen gefunden werden.

Doch wie steht es um die tatsächliche Ungleichheit in den USA heute? Einkommensungleichheit in den USA hat vielfältige Ursachen, die sich jahrzehntelang gegenseitig verstärkt haben. Thomas Piketty und Emmanuel Saez zeigen, dass vor gut hundert Jahren die Ungleichheit in Europa stärker ausgeprägt war als in den USA, sich dies aber heute ins Gegenteil verkehrt hat (Piketty und Saez 2014, S. 838). Spätestens seit dem Ende der 70er-Jahre ist klar, dass sich die Schere zwischen hohen, mittleren und geringen Einkommen vergrößert. Bei Vermögen ist der Trend noch stärker. Dabei kann man sagen, dass die hohen und sehr hohen Einkommen weitergewachsen sind, während die Einkommen aller anderen Einkommensgruppen stagnieren. Seit dem Ende der 70er-Jahre sind die durchschnittlichen Einkommen praktisch nicht von der Stelle gekommen (Kearney et al. 2015). Paul Krugman schätzt aber, dass seit den 70er-Jahren die Einkommen der Top 1 % um 165 % gestiegen sind und die der Top 0,1 % sogar um über 360 % (2017, S. 68). In den USA wurde lange ein gewisses Maß an Ungleichheit als Teil einer dynamischen Wirtschaft akzeptiert. Hier scheint in den letzten Jahren ein Umdenken stattgefunden zu haben. Es hat sich eine gewisse Ablehnung und Skepsis innerhalb der amerikanischen Bevölkerung ausgebreitet, die ihre Teilhabe am wirtschaftlichen und politischen Leben schwinden sieht. Zusätzlich macht es die extreme Polarisierung zwischen der Demokratischen und Republikanischen Partei fast unmöglich, eine kohärente nationale Agenda zur Bekämpfung von ökonomischer Ungleichheit und strukturellen Problemen aufzustellen, geschweige denn sie umzusetzen.

Weiterhin hat sich die Wahrnehmung von Ungleichheit und wirtschaftlicher Sicherheit in den letzten Jahren verändert und so zunehmend zum Verlust von Vertrauen in die etablierte Politik und ihre Institutionen geführt. Bürger:innen nehmen Ungleichheit verstärkt in ihrem persönlichen Kontext wahr. Sie empfinden das gesamte politische und wirtschaftliche System als unfair und von ihrer Lebenssituation entfremdet. Der Verlust von Vertrauen in die etablierte Politik wird sowohl von der wahrgenommenen als auch der real anwachsenden Ungleichheit begünstigt. Die amerikanische Bevölkerung hat nicht mehr das Gefühl, von der Politik repräsentiert und ernst genommen zu werden, und dadurch Angst vor wirtschaftlichem Abstieg (Lammert und Vormann 2019). Kommunikation spielt dabei eine zentrale Rolle: „individual perception – distrust, as it were – is strongly influenced by public discourses, dominant ideologies and the ways in which specific policy questions are framed (e.g. in the media)“ (Lammert und Vormann 2019, S. 140). Das Misstrauen in etablierte Institutionen hängt folglich auch davon ab, wie Ungleichheit in der Öffentlichkeit dargestellt wird. Vertrauen und Unterstützung von politischer Seite und etablierten Institutionen kann nur hergestellt werden, wenn die Bürger:innen den Eindruck haben, eine faire Chance zu bekommen: „the individual actor is showing support for the system (voting for established political actors, paying taxes etc.) as long as he or she has the impression of being treated fairly, and benefitting in one way or another from the political system and the economic order“ (Lammert und Vormann 2019, S. 153). Dabei sind vor allem *equality of opportunity*, wirtschaftliche Sicherheit und ein für die Nöte und Sorgen der Bevölkerung empfänglicher, sichtbarer Staat wichtig.

**Figure 1:**
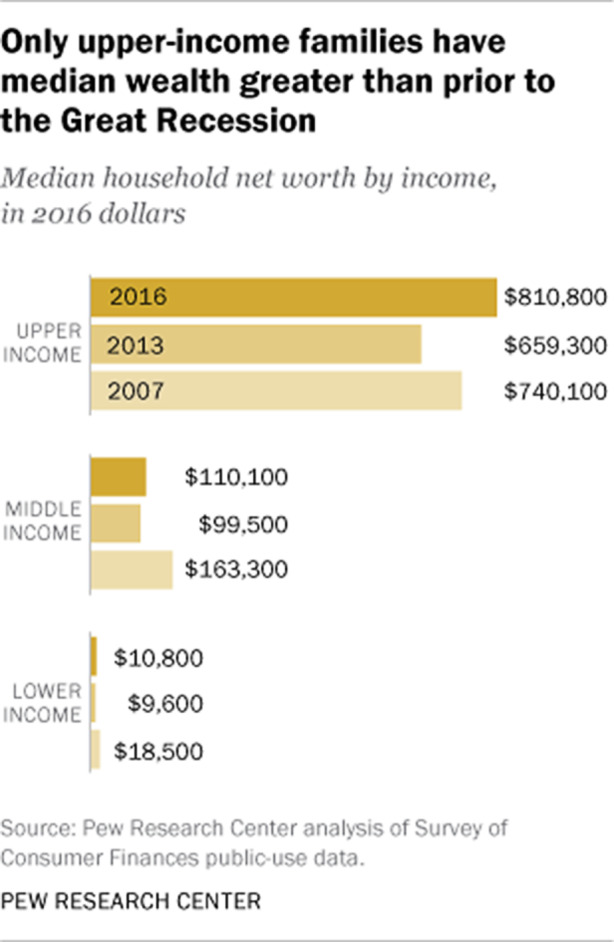

Doch die Vorstellung von Chancengleichheit und Fairness ist vor allem seit der Wirtschaftskrise von 2008 und der Rettung des Bankensektors, welcher die Krise erst verursacht hatte, schwer erschüttert (Hacker und Pierson 2010, S. 153). Diese Wahrnehmung hat sich auch fast zehn Jahre später nicht geändert, denn die Besitzverhältnisse der unteren und mittleren Einkommensgruppen haben sich immer noch nicht erholt (siehe Abb. 1).

**Figure 2:**
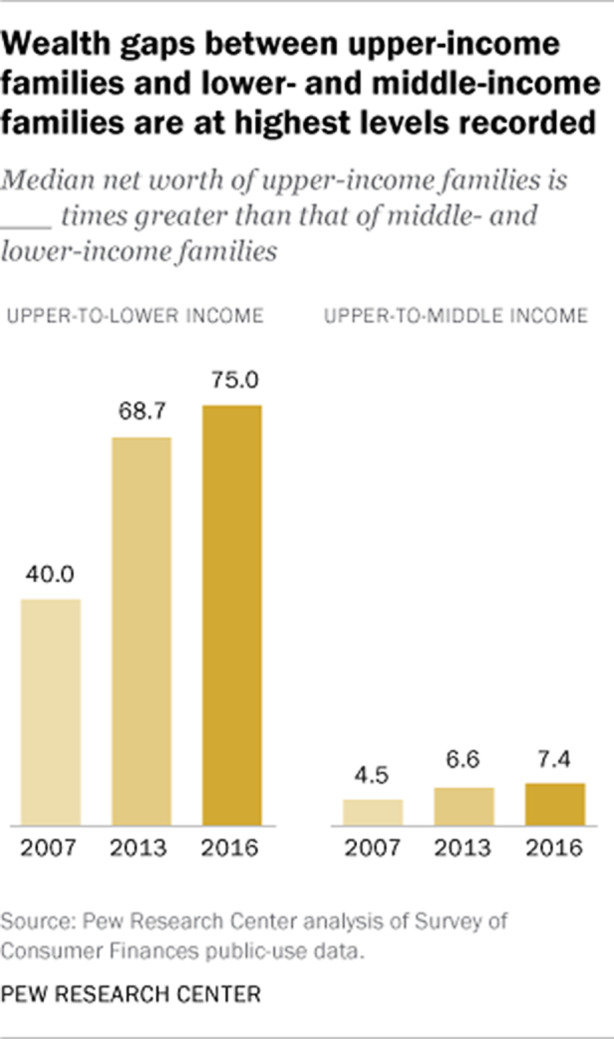

Die Schere zwischen den unteren und mittleren Einkommen einerseits und den hohen Einkommen andererseits ist so groß wie nie (siehe Abb. 2). 2016 war der *median net worth* der Topverdiener:innen 75-mal so hoch wie der Durchschnittswert eines Geringverdienerhaushalts und 7,4-mal so hoch wie der eines Mittelschichtshaushalts.

Die weltweite Covid-19 Pandemie hat die USA nun erneut in eine tiefe wirtschaftliche Krise gestürzt, die den Haushalten, die sich langsam von der Finanzkrise erholt hatten, abermals schweren Schaden zufügt (Pew-Research-Center 2020; NYT 2021).

Infolge der wirtschaftlichen Probleme und der größer werdenden Einkommensschere ist es nicht verwunderlich, dass immer mehr Amerikaner:innen den Status quo nicht länger akzeptieren wollen und den Glauben an die Möglichkeit ihres wirtschaftlichen Aufstiegs verloren haben.

## Ungleichheitsnarrative der politischen Linken: Die Millennial-Generation, Elizabeth Warren und das *rigged system*

Nach der Wahl Donald Trumps 2016 und den Wahlen 2020 wird oft Trumps populistische Rhetorik und ein Abdriften der USA in nationalistische und fremdenfeindliche Ideologie diskutiert. Doch der Gegentrend ist in den zutiefst gespaltenen USA ebenfalls zu beobachten: Der Aufstieg einer neuen linken Opposition, die sich vor allem in der jüngeren Generation, etwa den Millennials,^4^ zu verfestigen scheint. Die Millennial-Generation ist die bisher gebildetste und ethnisch vielfältigste Generation der US-Geschichte (Milkman 2017, S. 2). Sie war von der letzten Wirtschaftskrise stark betroffen und kämpft weiterhin darum, eine wirtschaftlich sichere Existenz aufzubauen (Ajilore 2019, S. 2).

**Figure 3:**
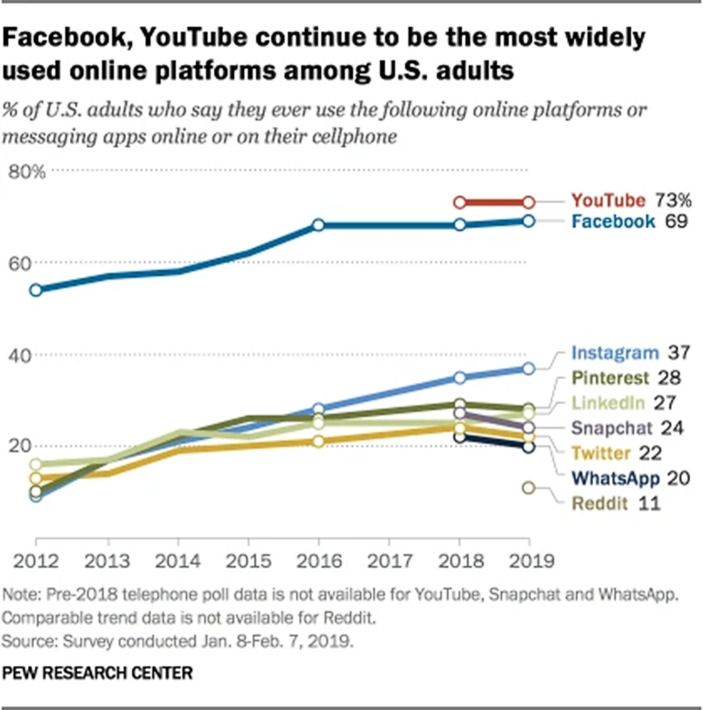

Politisch aktiv ist die Millennial-Generation zu verschiedenen Themen, etwa in der *Black Lives Matter*-Bewegung oder dem *Women’s March*. Doch scheint auch das kapitalistische System selbst immer kritischer gesehen zu werden. Diese Generation, die in der Internet-Ära erwachsen wurde, nutzt verstärkt digitale Kommunikation und Online-Plattformen (Milkman 2017, S. 2), auch für politischen Protest und Aktivismus (Nielsen 2013, S. 175). Eine Studie aus dem Jahr 2013 zeigt, dass *digital activism* auf bestimmte Plattformen zurückgreift. Dabei sind *Facebook* und *Twitter* sowie *YouTube *für Videocontent entscheidend (siehe Abb. 3) (Edwards et al., in Rauscher 2018, S. 201). Es werden auch zunehmend visuelle Möglichkeiten zur Kommunikation auf Plattformen wie Instagram^5^ genutzt. Die visuell geprägten Plattformen haben inzwischen text-basierte Plattformen wie *Twitter* teils in ihrer Userstärke und Verweildauer abgelöst (Russman und Svensson 2016, S. 1).

Themen wie ökonomische Ungleichheit und eine kritische Haltung gegenüber den Exzessen des kapitalistischen Systems scheinen in den letzten Jahren durch die Millennial-Generation und deren Unterstützung linker Kandidat:innen verstärkt Eingang in den politischen *offline-* und *online*-Diskurs in den USA zu finden (Purdy 2017, S. 28). Dies sind Themen, die auch Elizabeth Warren aufgreift. Warren gehört damit klar dem linken Flügel der Demokratischen Partei an.

Als Außenseiterin in den Wahlkampf 2020 gestartet, wurde sie zwischenzeitlich zu einem der *frontrunner* der Demokratischen Kandidatensuche für das Präsidentenamt. *The Daily Beast* schrieb schon früh über sie: „Warren has been one of the most articulate voices challenging the excesses of Wall Street. Still, she enjoys an outsize celebrity for an academic and bureaucrat: a favorite guest of Jon Stewart, Warren, 62, has become a hero of the left, a villain to the right, and a fascination for everyone in between“ (Jacobs 2011). 2011, als Warren ihre politische Karriere begann, machte gerade ein Zeltlager mitten in Manhattan weltweit auf sich aufmerksam: *Occupy Wall Street*. Die Zeit schien reif für eine linke Kandidatin wie Warren, die bald nationale Aufmerksamkeit genoss. Sie sprach über Themen wie Ungleichheit, die Macht von Wall Street und *Big Money* in der Politik sowie die immer größer werdenden Belastungen für die Mittelschicht. Dass sie bereits vor ihrer politischen Karriere in diesen Feldern gearbeitet hatte verlieh ihr eine gewisse Glaubwürdigkeit (Walker 2014), genauso wie ihr Abstimmungsverhalten im Senat. Laut Progressive Punch hat sie in 98,95 % aller Abstimmungen als Senatorin progressiv gewählt (ProgressivePunch 2019). Mit diesen Voraussetzungen schienen Unterstützer sie lange Zeit als authentische Figur im Wahlkampf wahrzunehmen.

Trotz Warrens letztendlichem Ausscheiden aus dem Wahlkampf 2020 lohnt es sich, einen Blick auf die Kampagne einer der prominentesten Vertreter:innen des linken Flügels der Demokratischen Partei zu werfen und festzustellen, wie hier progressive Ziele strategisch kommuniziert werden. Warren zielt darauf ab, komplexe Zusammenhänge verständlich darzustellen und dabei zugänglich zu wirken. Sie scheint in den Augen vieler über die Jahre ihrer Kernbotschaft treu geblieben zu sein, wodurch sie in ihren Forderungen authentischer wirkte (Walker 2014, S. 4). Nach dem Wahlkampf 2020 hat sie weiterhin eine loyale Unterstützergruppe hinter sich (Goldmacher und Herndon 2020). Ihre Message und ihre Rhetorik haben Wiedererkennungswert. So sprach sie bereits 2012 beim Parteitag der Demokratischen Partei (DNC) von einem *rigged system*, das der Mittelschicht keine Chance lasse (Warren 2012a). Eine ihrer bis heute viel genutzten Phrasen, *leveling the playing field*, benutzte sie ebenfalls bereits 2012 bei ihrer DNC Rede: „if we have the chance to fight on a level playing field, where everyone pays a fair share and everyone has a real shot then no one can stop us!“ (Warren 2012a). *Leveling the playing field* ist kein Ausdruck, den Warren erfunden hat, sondern eine im Englischen geläufige Umschreibung und Metapher aus dem Sport, um Fairness und Chancengleichheit auszudrücken. Es ist einer ihrer über die Jahre wiederkehrenden Ausdrücke. In ihrer Victory Speech 2012 nach den Senatswahlen sagte sie: „… we’re going to fight for a level playing field and we’re going to put people back to work“ (Warren 2012b).

In ihrer Rede nach der Wiederwahl 2018 zur Senatorin sprach sie zwar nicht von *leveling the playing field*, doch sie sprach wieder vom *rigged system*, das große Teile der amerikanischen Bevölkerung von Teilhabe in Politik, Gesellschaft und Wirtschaft ausschließt: „We came to this movement to pick a fight – a fight on behalf of millions of Americans who are getting ripped off in a rigged economy and ignored by a corrupt government in Washington“ (DeCosta-Klipa 2018). Kurz darauf kritisierte sie im Frühjahr 2019 in der Rede, die sie zur formellen Ankündigung ihrer Präsidentschaftskandidatur nutzte: „Today, millions and millions and millions of American families are also struggling to survive in a system that’s been rigged, rigged by the wealthy and the well-connected“ (Taylor 2019). Hier vermittelte sie nochmals, dass sie als Präsidentin für *structural change* stehen würde. In einer kurzen Stellungnahme nach ihrem Rückzug aus dem Präsidentschaftswahlkampf 2020 ging sie erneut auf die Anfänge ihrer politischen Karriere 2012 ein:

10 years ago I was teaching a few blocks from here and talking about what was broken in America and ideas for how to fix it. And pretty much nobody wanted to hear it. And I’ve had a chance to get out there and talk with millions of people and we have ideas now that we talk about that we just weren’t talking about even a year ago, a 2 cent wealth tax and universal childcare (…) and canceling student loan debt (…). Those are life changing events for people. (…). (Warren 2020a)

Hier betont Warren, dass viele der Inhalte, mit denen sie ihren Wahlkampf bestritt, mittlerweile immer mehr zum Mainstream gehören, zumindest in ihrer Partei, und Ideen wie eine Vermögenssteuer oder der Abbau von Studienschulden (ein Thema, mit dem sie sich seit ihren Anfängen beschäftigte) nicht mehr nur von politischen Außenseitern:innen diskutiert werden.

Eine Vermögenssteuer, für die sich Warren und Konkurrenten wie Bernie Sanders im Wahlkampf stark machten, wurde im Frühjahr 2020 heftig diskutiert, bevor die Corona-Pandemie Debatten über andere politische Inhalte, darunter auch mögliche Steuererhöhungen, vorerst ein Ende setzte. Trotzdem bleibt das Thema relevant, denn eine Steuer, die die Reichsten stärker in die Verantwortung nehmen würde, könnte in den USA eine große re-distributive Maßnahme einleiten, um die Mittelschicht zu stärken. Eine solche Maßnahme würde die wachsende Ungleichheit in der amerikanischen Bevölkerung stärker und direkter adressieren. Außerdem würde sie der Finanzierung weitereichender sozialer Programme wie gebührenfreien Colleges, Kinderbetreuung, einer Infrastrukturreform oder einer allgemeinen Krankenversicherung dienen.^6^ Warren und große Teile des linken Flügels ihrer Partei sehen eine solche Reform als eine Lösung an, die steigende Ungleichheit (vor allem bezüglich Vermögen) in den USA auszubalancieren, die Macht der Superreichen in der Politik zu reduzieren und strukturelle Probleme zu lösen (Rappeport und Kaplan 2019). Warren betont konstant strukturelle Schwächen im amerikanischen System, die dem *American Dream* und vor allem der Chancengleichheit (*equality of opportunity*) und Fairness fundamental entgegenstehen. Alle Amerikaner:innen sollten ihren *fair shot* erhalten und sich einen Weg in die Mittelschicht erarbeiten können. Progressive Politiker:innen wie Warren setzen sich daher als Antidote zum vorherrschenden politischen Establishment in Szene.

## Kommunikationsstrategie Warrens

Warren ist zwar selbst schon über 70, doch ist ihre politische Kommunikation auf eine junge Zielgruppe zugeschnitten. Sie ist ein Social Media Schwergewicht geworden, das unter den Kandidat:innen der Demokratischen Partei im Vorwahlkampf 2019/2020 nur von Bernie Sanders übertroffen wurde. Warren nennt heute über 3,4 Mio. Follower auf Facebook, 5,4 Mio. auf Twitter und 2,6 Mio. auf Instagram ihr Eigen. Bernie Sanders folgen 5,7 Mio. Menschen auf Facebook, 14,4 Mio. auf Twitter und 6,1 Mio. auf Instagram. Im Vergleich dazu waren die anderen Bewerber:innen der Demokratischen Partei im Vorwahlkampf abgeschlagen^7^.

Auf den Social Media-Seiten von Warren geht es darum, ihre Follower direkt anzusprechen, mitzunehmen und zu zeigen, wie sie auf dem Campaign Trail mit ihrem Publikum interagiert. Daher werden auf Facebook^8^ oder Twitter^9^ häufig *Townhalls* oder kurze Videos veröffentlicht, in denen sie auf der Bühne spricht oder Unterstützer:innen trifft. Eines der beliebtesten Events und der geteilten Beiträge waren die Selfie-Lines und sogenannten *pinky promises* unter dem Motto „I’m running for president because that’s what girls do.“, die sie Mädchen gab, die zu ihren Townhall Meetings kamen (Goldmacher und Herndon 2020). Auf Twitter wurden außerdem kurze Statements als Tweets abgesetzt, oft zu aktuellen Ereignissen, sowie Videos gepostet, in denen Warren z. B. *pinky promises* gab oder Spender:innen anrief. Diese waren meist sehr überrascht, die echte Elizabeth Warren am Telefon zu haben. Solche Videos aus dem ‚echten Leben‘ werden von Warrens Team auf allen Plattformen geteilt. Auf Instagram^10^, wo bildlicher Content wichtiger ist als auf Twitter, setzte Warren auf Infografiken sowie direkte Links, die auf ihre Website führen. Dort kann man nach wie vor ihre Pläne genau nachlesen und es gibt interaktive Angebote wie den *Student Debt Calculator*. Dadurch, dass Warren diese Plattformen benutzt, nähert sie sich dem jungen Publikum an, das mit diesen vertraut ist und dort Inhalte und Formate wie etwa *Instagram Stories* nachfragt. Sie schlägt hier aber bewusst leichtere Töne an, macht sich nahbarer und erweckt so den Anschein, ihre Follower seien direkt auf dem Campaign Trail dabei. Dies ist für erfolgreiche Online-Kommunikation entscheidend, ohne die ein Wahlkampf heute undenkbar ist. Zudem verlinkt sie von allen Social Media-Plattformen auf ihre Website, auf der sie zu vielen Kernthemen des Wahlkampfs in die Tiefe geht.

In ihrer Kommunikation stellt Warren die Themen Chancengleichheit und Fairness in den Mittelpunkt. Außerdem soll gezeigt werden, welche Pläne Warren bereits ausgearbeitet hat. Damit setzte sie im Wahlkampf einen Gegenpol zu Präsident Trump, der zu wenigen Themen eine kohärente Strategie aufzuweisen hatte. Im Folgenden wird genauer auf Warrens Pläne und die darin kommunizierten Narrative eingegangen und diese analysiert. Dadurch wird exemplarisch strategisches Framing progressiver Botschaften zu ökonomischer Ungleichheit aufgezeigt.

### Narrative und strategisches Framing

Die auf Warrens Internetseite^11^ vorgestellten politischen Forderungen stehen unter dem Motto: „What will Elizabeth Warren do?“ Insgesamt wurden im Vorwahlkampf 48^12^ spezifische Pläne in verständlichen Worten erklärt. Unterstützer:innen hatten die Möglichkeit, diese zu unterschreiben und Warrens Botschaften so direkt auf Social Media zu teilen. Auf der Website waren die politischen Themen in fünf Schwerpunkte eingeteilt: *Strengthening our Democracy, Rebuild the Middle Class, Equal Justice under Law, End Washington Corruption* und *A Foreign Policy for all*. Bereits bei der Formulierung dieser Kategorien spielen unterschiedliche Aspekte ökonomischer Ungleichheit eine Rolle. Es wird der Eindruck vermittelt, die Mittelschicht sei geschwächt, vor dem Gesetz seien einige ‚gleicher als andere‘, die Regierung sei korrupt und repräsentiere nicht mehr die gesamte Bevölkerung.

Warren bediente sich dabei strategischer Frames, um Schwerpunkte zu setzen und Bewertungen vorzuschlagen, und verband ihr Narrativ der ökonomischen Ungleichheit und der Versprechen des *American Dream* so mit dem weiteren öffentlichen Diskurs. Wie in der Einleitung angedeutet werden Narrative häufig von politischen Akteur:innen genutzt, um ihren Ansätzen Legitimität zu verleihen (Yildiz et al. 2018) und ihre Agenda durch bekannte ‚Erzählungen‘ in nachvollziehbarer oder identitätsstiftender Weise zu kommunizieren. Politische Narrative werden so zu performativen ‚Sprechakten‘ politischer Entscheidungsträger:innen (Gadinger et al. 2014, S. 7). Narrative können auch als eine Form persuasiver Kommunikation von Akteur:innen eingesetzt werden, welche nur beschränkten Zugang zu technischen, wissenschaftlichen oder fachlichen Diskursen (Polletta 1998a, b) oder traditionellen Kommunikationswegen haben (Naples 2003). Diese (Rand‑)Gruppen nutzen Storytelling, indem persönliche Erfahrungen kommuniziert und somit vermeintlich „unangreifbare“ Tatsachen in den Diskurs eingebracht werden sollen (Nepstad 2002; Polletta 1998a).

Frames unterstützen Narrative, indem sie die persönlichen Geschichten mit größeren Diskursen verbinden und Interpretationen vorgeben. Strategisches Framing gibt den politischen Problemen der Narrative einen sprachlichen Rahmen und schreibt ihnen eine Bedeutung zu (Gadinger et al. 2014; Hauke 2014).^13^ Es geht dabei darum, Themen in einer bestimmten Weise darzustellen und im öffentlichen Diskurs und den Medien zu platzieren. Frames finden sich in der strategischen Kommunikation von Politiker:innen, die eine bestimmte Interpretation eines Themas vorschlagen, in der Auswahl und Anpassung dieser Interpretation durch Medienvertreter:innen und in der Rezeption dieser Frames durch Teilnehmende eines Diskurses (Matthes 2012, S. 249 f.). Sogenannte Kommunikations-Frames können gezielt konstruiert werden, um einen Teil eines Sachverhalts in einen bestimmten Zusammenhang zu setzen, dadurch den Schwerpunkt eines Diskurses zu verschieben und somit zu einer spezifischen Problembewertung durch ein bestimmtes Publikum zu führen (Oswald 2019, S. 23–24). Framing kann folglich strategisch eingesetzt werden. Kommunikations-Frames basieren darauf, dass in jeder Kommunikation einige Aspekte ausgewählt und betont, andere hingegen weggelassen werden: „To frame is to *select some aspects of a perceived reality and make them more salient in a communicating text.*“ [Hervorhebung im Original] (Entman 1993, S. 52) Basierend auf dieser Definition sind Frames laut Robert Entman (1993) durch vier Elemente gekennzeichnet:

- Problemdefinition
- Ursachenzuschreibung
- Problembewertung basierend auf moralischen oder anderen Werten
- Handlungsempfehlung zur Lösung dieser Probleme

Es müssen allerdings nur mindestens zwei dieser Elemente in einer Botschaft vorliegen, damit von Framing gesprochen werden kann.

Der Erfolg von strategischem Framing hängt davon ab, inwieweit Frames dem Glaubenssystem des Publikums entsprechen. Die Voraussetzung für eine erfolgreiche Diskursverschiebung mit Hilfe von Kommunikations-Frames ist, dass die hervorgehobenen Aspekte mit dem *belief system *eines Individuums übereinstimmen (Oswald 2019, S. 23–24). Gerhards und Rucht beschreiben *belief systems *als „a configuration of ideas and attitudes in which the elements are bound together by some form of constraint or functional interdependence“ (1992, S. 575). Die einzelnen Elemente dieser Glaubenssysteme sind die Basis für individuelle Einstellungen hinsichtlich politischer Institutionen und Ideologien und beeinflussen somit auch, wie Individuen politische Themen interpretieren. Studien zeigen, dass Menschen leichter politische Botschaften akzeptieren, die in Einklang mit ihrem Wertesystem stehen (Druckman und Lupia 2016; Sabatier 1998). Nachrichten, die hingegen konträr zu den Werten eines Individuums sind, werden ausgeblendet oder sogar abgelehnt (Landau et al. 2014; Entman 1993). Die Resonanz von strategisch genutzten Frames hängt folglich davon ab, inwieweit diese mit der Weltanschauung der Zielgruppe übereinstimmen.

Bei der Überzeugungskraft eines Frames spielen auch die Kommunikator:innen eine Rolle. Beide Konzepte, Narrative und strategische Frames, zielen auf Aktion ab, da sie ein Publikum – zum Beispiel innerhalb einer Bewegung oder Partei – mobilisieren wollen. Dafür heben soziale Bewegungen, Interessensgruppen oder Politiker:innen eine bestimmte Sichtweise auf ein Thema hervor. Anders als Narrative werden Kommunikations-Frames aber meist von Akteur:innen eingesetzt, die durch eine Form von institutioneller Autorität legitimiert sind (Olsen 2014, S. 251). Die Glaubwürdigkeit der Person, die diese Frames nutzt, kann deshalb die Resonanz der von ihnen kommunizierten Frames beeinflussen. Dies umfasst beispielsweise, ob Frames konstant eingesetzt werden, ob das Gesagte mit dem Verhalten der Kommunikator:innen übereinstimmt oder wie valide Daten und Fakten sind, die einen Frame stützen (Olsen 2014, S. 251; Benford und Snow 2000, S. 621). Der Erfolg eines Frames hängt demnach davon ab, wie viel Gewicht ihm Expertise und Verhalten der Kommunikator:innen oder Forschungsergebnisse verleihen.

### Frame-Analyse: Vorgehen und Methode

Um zu identifizieren, welche Frames Elizabeth Warren zu ihrem Narrativ rund um den *American Dream *kommuniziert und welche Rolle dabei Ungleichheit spielt, wurde eine Korpus-Analyse durchgeführt. Der Korpus aus über 120.000 Wörtern^14^ setzt sich aus den ausformulierten Plänen auf Warrens Website im Rahmen ihrer Wahlkampagne zusammen. Bei einer reinen Frequenzanalyse sind die häufigsten Wörter in einem Korpus meist Funktionswörter, im Englischen beispielsweise „and“, „the“ oder „a“. Diese sagen jedoch wenig über die Inhalte eines Textkorpus aus. Daher wurden im zweiten Schritt häufig auftretende Begriffe, die keine Funktionswörter sind, thematisch gruppiert, um sie für eine qualitative Inhaltsanalyse auszuwerten. Die Software-gestützte Analyse ergab^15^, dass einerseits Aspekte rund um Reichtum, Großkonzerne und das politische System und andererseits das Thema Fairness in Warrens Diskurs prominente Positionen einnehmen. Eine überraschende Erkenntnis der Analyse war, dass die Senatorin die Begriffe *equality *und *inequality* nur sparsam verwendet, obwohl (ökonomische) Ungleichheit einer ihrer Schwerpunkte ist. Statt *in-/equality* spricht Warren vor allem über Ungleichheit im Sinne von *equality of opportunity*, also Fairness und Chancengleichheit. Hier wird sichtbar, dass es notwendig ist, die untersuchten Aussagen auch qualitativ zu bewerten. Nur, weil die Begriffe *equality/inequality* nicht sehr häufig vorkommen, heißt das nicht, dass nicht trotzdem über diese Problematik gesprochen wird.

Im nächsten Schritt wurde der Fokus deshalb auf die Themen Fairness sowie Reichtum und Macht gelegt. Eine Kollokations-Analyse^16^ der Schlagworte *fair *und *wealth *zeigte, welche Begriffe im Zusammenhang mit diesen Themen verwendet werden. Die Ergebnisse der Kollokations-Analyse, einschließlich Frequenz der Begriffe im Korpus, MI Score^17^, Beispiele für ihre Nutzung sowie die Information, in welchen Plänen auf Warrens Website sie auftauchen, zeigen Tab. 1 und 2.

**Table 1:** 

Absolute Frequenz^a^	MI Score	Kollokation	Beispiel (Wahlkampfplan)	Kontext – Wahlkampfpläne auf Warrens Website
11	9.16519	Share	„My plan brings our Social Security system back into balance by asking the top 2 % of earners to start contributing a fair share of their wages to the system […]“ (Expanding Social Security)	Empowering American Workers and Raising Wages; Ending the Opioid Crisis; Expanding Social Security; Real Corporate Profits Tax; Tackling the Climate Crisis Head On; Ultra-Millionaire Tax
9	7.00467	Farmers	„His [Anm. d. Autors: FDR’s] administration set up a system that guaranteed farmers fair prices, tackled overproduction, and reversed environmental degradation.“ (A New Farm Economy)	A New Farm Economy
7	8.85415	Price	„Just like workers need a living wage, farmers need a fair price […]“ (A New Farm Economy)	A New Farm Economy; A New Approach to Trade ; Investing in Rural America; Tackling the Climate Crisis Head On
5	9.46825	Shot	„**The Small Business Equity Fund is another tool we should use. It will create jobs, spur economic growth, and move us closer to an America where everyone has a fair shot to succeed.**“ (Leveling the Playing Field for Entrepreneurs of Color)	How We Can Break Up Big Tech; Leveling the Playing Field for Entrepreneurs of Color; Universal Child Care; Valuing the Work of Women of Color
4	8.70575	Judiciary	„They [Anm. D. Autors: reforms] will also hold the vast majority of judges who act in good faith to the highest ethical standards, and in the process, begin to restore accountability and trust in a fair and impartial federal judiciary.“ (Restoring Trust in an Impartial and Ethical Judiciary)	Restoring Trust in an Impartial and Ethical Judiciary
4	8.36872	Contribute	„We should be increasing Social Security benefits and asking the richest Americans to contribute their fair share to the program.“ (Expanding Social Security)	Expanding Social Security
3	10.95368	Achievable	„***Provide a fair and achievable pathway to citizenship.***“ (A Fair and Welcoming Immigration System)	A Fair and Welcoming Immigration System
3	10.53864	Minded	„Our judiciary only functions properly when it lives up to this promise, and it risks eroding its legitimacy when the American people lose faith that judges are ethical and fair-minded.“ (Restoring Trust in an Impartial and Ethical Judiciary)	End Washington Corruption; Restoring Trust in an Impartial and Ethical Judiciary
3	9.95368	Scheduling	„Federal contractors must extend a $ 15 minimum wage and benefits (including paid family leave, fair scheduling, and collective bargaining rights) to all employees.“ (Valuing the Work of Women of Color)	Fighting For Justice As We Combat The Climate Crisis; Leading in Green Manufacturing; Valuing the Work of Women of Color
3	9.36872	Overproduction	„We need to replace our failed system with a tried-and-true method that guarantees farmers that fair price and ends overproduction.“ (A New Farm Economy)	A New Farm Economy
3	9.21672	Guarantees	„**And I’ll take it one step further – charting a new farm economy that replaces our government’s failed approach with one that guarantees farmers a fair price** […]“ (Investing in Rural America)	A New Farm Economy; Investing in Rural America
3	7.78376	Leave	„For instance, my Green Manufacturing plan makes a $ 1.5 trillion procurement commitment to domestic manufacturing contingent on companies providing fair wages, paid family and medical leave, fair scheduling practices, and collective bargaining rights.“ (Fighting For Justice As We Combat The Climate Crisis)	Fighting For Justice As We Combat The Climate Crisis; Leading in Green Manufacturing; Valuing the Work of Women of Color

^a^*N* = 121.324

**Table 2:** 

Absolute Frequenz^a^	MI Score	Kollokation	Beispiel (Plan)	Kontext – Wahlkampfpläne auf Warrens Website
11	9.80612	Gap	**„The small business gap is another example of how the racial wealth gap in America holds back our economy and hurts Black, Latinx, Native American, and other minority families and communities.**“ (Leveling the Playing Field for Entrepreneurs of Color)	A New Farm Economy; Affordable Higher Education for All; Leveling the Playing Field for Entrepreneurs of Color; Safe and Affordable Housing
8	10.14316	Racial	„But we expect everyone but the wealthy to take on mountains of debt if they want to get a post-secondary education. This is closing off opportunities for generations of Americans and widening this country’s racial wealth gap.“ (Affordable Higher Education for All)	Affordable Higher Education for All; Leveling the Playing Field for Entrepreneurs of Color; Safe and Affordable Housing
5	7.27007	Black	„An economic analysis from leading experts on student loan debt finds that **my plan would provide at least some debt cancellation for 95** **% of people with student loan debt** […]**, substantially increase Black and Latinx wealth, and help close the racial wealth gap**.“ (Affordable Higher Education for All)	Affordable Higher Education for All; Leveling the Playing Field for Entrepreneurs of Color; Safe and Affordable Housing
5	7.93166	Build	„My plan will end the policies that have perpetuated this [Anm. D. Autors: farmers of color’s] discrimination and help rural families of color build wealth and sustainable livelihoods.“ (A New Farm Economy)	A New Farm Economy; Comprehensive Criminal Justice Reform; Fighting For Justice As We Combat The Climate Crisis
4	7.46035	White	„On average, Black, Latinx, Native American, and other minority households have a lot less wealth than white households.“ (Leveling the Playing Field for Entrepreneurs of Color)	Affordable Higher Education for All; Leveling the Playing Field for Entrepreneurs of Color; Safe and Affordable Housing
4	8.69065	Household	„The Fund will provide no-strings-attached grants to entrepreneurs eligible for the Small Business Administration’s existing 8(a) program and who have less than $ 100,000 in household wealth. That wealth threshold is roughly the national average, but it’s over ten times the estimated median net worth of Native American families.“ (Honoring and Empowering Tribal Nations and Indigenous Peoples)	Honoring and Empowering Tribal Nations and Indigenous Peoples; Leveling the Playing Field for Entrepreneurs of Color
4	8.93166	Latinx	„[…] I’ve proposed an expansion of the Community Reinvestment Act to ensure that mortgage lenders in communities of color lend to everyone on an equal basis, a student debt plan that invests $ 50 billion in HBCUs and Minority-Serving Institutions and substantially increases Black and Latinx wealth, and a historic new down payment assistance program […]“ (Leveling the Playing Field for Entrepreneurs of Color)	Affordable Higher Education for All; Leveling the Playing Field for Entrepreneurs of Color; Safe and Affordable Housing
4	9.58373	Threshold	„It [Anm. D. Autors: a Small Business Equity Fund] will be targeted at closing the entrepreneurship gap by limiting grants to entrepreneurs who are eligible for the Small Business Administration’s existing 8(a) program and who have less than $ 100,000 in household wealth. That wealth threshold is roughly the national average, but it’s five times the median net worth of Latinx and Black families, and over ten times the median net worth of Native American families.“ (Leveling the Playing Field for Entrepreneurs of Color)	Honoring and Empowering Tribal Nations and Indigenous Peoples; Leveling the Playing Field for Entrepreneurs of Color
3	7.02185	Less	„On average, Black, Latinx, Native American, and other minority households have a lot less wealth than white households.“ (Leveling the Playing Field for Entrepreneurs of Color)	Honoring and Empowering Tribal Nations and Indigenous Peoples; Leveling the Playing Field for Entrepreneurs of Color
3	7.65412	Close	„Elizabeth’s housing plan will lower rents by 10 %, help close the racial wealth gap, and create 1.5 million new jobs.“ (Safe and Affordable Housing)	Affordable Higher Education for All; Safe and Affordable Housing

^a^*N* = 121.324

Basierend auf den Ergebnissen wurde der Kontext, in dem die Kollokationen auftreten, genauer betrachtet, um so Argumentationsmuster und damit von Warren verwendete Frames zu identifizieren. Dies wird in der nachfolgenden Frame-Analyse sichtbar.

### Der *fairness*-Frame

Einer der prominentesten Frames, den Warren in ihrer Kommunikation einsetzt, ist der *fairness*-Frame. Dieser gibt eine Interpretation und Bewertung des Zustands der USA vor, der dem *American Dream *nicht mehr gerecht wird. Warren spricht z. B. von „fair pay“ oder „fair share“. Der *fairness-*Frame beinhaltet die folgenden Elemente:

- **Problemdefinition**: (Fehlende) ökonomische Wertschätzung bestimmter Bevölkerungsgruppen.
  Diese manifestiert sich in inadäquater Bezahlung für Arbeit oder Produkte. Aussagen wie „we owe them a fair pay for the work“ (Warren 2019g) und „farmers need a fair price, one that covers the costs“ (Warren 2019e) fordern mehr Anerkennung für erbrachte Arbeit in Form von höheren Löhnen und Preisen.
- **Ursachenzuschreibung**: Korruption und Vorteile für die Oberschicht bestehend aus den Superreichen, Großkonzernen und Politiker:innen führen zu ökonomischer Ungleichheit.
  Mit Forderungen wie „It’s time Washington stopped trying to slash Social Security benefits for people who’ve earned them“ (2019c) und „It’s time to break up big Ag[riculture] and guarantee farmers a fair share“ (2019e) macht Warren klar, wer aus ihrer Sicht für die ungleiche Einkommensverteilung verantwortlich ist und hart arbeitende Teile der Bevölkerung in wirtschaftlicher Unsicherheit zurück lässt.
- **Problembewertung basierend auf moralischen oder anderen Werten**: Unfaires System.
  Das unfaire amerikanische System (Wirtschaft, Politik, Gesellschaft) wird von der Oberschicht ausgenutzt. Ob in wirtschaftlicher Hinsicht von „employers who engage in unfair labor practices“ (Warren 2019a) oder im Bildungssystem, „preventing our higher education system from fairly serving lower-income students and students of color“ (Warren 2019f). Das *rigged system* ermöglicht es Großkonzernen oder der Regierung, die faire Bezahlung und Anerkennung harter Arbeit zu umgehen und die ungleiche Chancenverteilung im Land zu vergrößern.
- **Handlungsempfehlung zur Lösung dieser Probleme**: Einkommensstarke Gruppen zur Verantwortung ziehen, um damit das Sozialsystem für einkommensschwache Gruppen zu verbessern sowie Chancen und mehr Partizipation zu schaffen.
  Forderungen nach mehr Fairness formuliert Warren in Aufrufen wie: „it’s time for the rich to pay their fair share“ (2019h). Sie fordert ein „America where everyone has a fair shot to succeed“ (2019d) mit dem Ziel: „make our economy and our democracy fairer and stronger“ (2019h).

Mit diesem Frame hebt Warren einen zentralen Punkt des *American Dream *hervor: Die gesellschaftliche und finanzielle Anerkennung harter Arbeit. Diese fehlt mittlerweile in den USA, nicht zuletzt aufgrund von systemischen Problemen. Mit dem Verweis auf dieses zentrale Narrativ der amerikanischen Gesellschaft und der Chancengleichheit werden fast alle Amerikaner:innen angesprochen, was eine breite Basis für die Resonanz des *fairness*-Frames schafft. Wo hinsichtlich Fairness und Chancengleichheit, oder *equality of opportunity*, relative Einigkeit in der Bevölkerung herrscht, ist man sich bei *equality of outcome* uneinig. Warren fordert aber, dass jeder eine faire Chance auf Erfolg bekommen sollte.

### Der *wealth*-Frame

In ihrer Kommunikation macht Warren außerdem klar, worin aus ihrer Sicht die Gründe für die aktuellen Missstände des unfairen Systems liegen und wer für diese verantwortlich ist. Sie nutzt dafür den *wealth*-Frame. Der Frame beinhaltet vier Elemente:

- **Problemdefinition**: Die *wealth gap* zwischen Arm und Reich
- **Ursachenzuschreibung**: Diskriminierende Regierungspolitik, egoistisches Verhalten der Wohlhabenden
- **Problembewertung basierend auf moralischen oder anderen Werten**: Ausbeutung der Arbeitnehmerschaft
- **Handlungsempfehlung zur Lösung dieser Probleme**: Gesetzesänderungen wie die *Ultra-Millionaire Tax*

Diese sollen im Folgenden ausführlicher analysiert werden.

- **Problemdefinition**
  In der Debatte um ökonomische Ungleichheit kommuniziert Warren die Vermögensunterschiede zwischen Arm und Reich als großes Problem. Mit Vergleichen wie „The 400 richest Americans currently own more wealth than all Black households and a quarter of Latino households combined“ (2019h) weist sie auf das Ausmaß der Ungleichheit hin, ohne direkt die Worte *equality* oder *inequality* zu nutzen. Ihr Gebrauch von „richest Americans“ und „wealth“ deutet aber darauf hin, dass sie die extreme Ungleichheit in den USA als großes Problem sieht. Die Situation der Haushalte, deren Wohlstand unter $ 100.000 liegt, hebt sie besonders hervor. Während dieser Betrag in etwa dem nationalen Durchschnitt entspricht, ist es „five times the median net worth of Latinx and Black families, and over ten times the median net worth of Native American families“ (Warren 2019d). Das heißt, die meisten Minderheiten in den USA sind wirtschaftlich schlechter gestellt.
  Unter der sich vergrößernden *wealth gap *leiden laut Warren nicht nur die weniger wohlhabenden Schichten, sondern die gesamte amerikanische Wirtschaft. Die Vermögenskonzentration und die mangelnde Besteuerung dieses Vermögens verhindern, dass der Regierung ausreichend Ressourcen zur Finanzierung von Infrastrukturprojekten, *Social Security* oder *Universal Child Care* zur Verfügung stehen. Dass sich viele Amerikaner:innen kein Studium mehr leisten können oder *non-white* (Klein‑)Unternehmer:innen nur bedingt gefördert werden verdeutlicht, „how the racial wealth gap in America holds back our economy“ (Warren 2019d). In Warrens Kommunikation ist die ungleiche Vermögensverteilung also auf individueller und gesamtwirtschaftlicher Ebene problematisch für die USA.
- **Ursachenzuschreibung**
  Dafür gibt es laut der Senatorin zwei Gründe: Eine langjährige Politik der Diskriminierung sowie die Gier der Reichen und des *corporate America*. Systemische Ungleichbehandlung, die immer noch nicht überwunden ist, nimmt vielen Amerikaner:innen die Möglichkeit, sich einen gewissen Wohlstand aufzubauen. Die Verantwortung dafür sieht Warren bei der Regierung, „because the government helped create that wealth gap with decades of sanctioned discrimination“ (2019d). Fehlende Chancen zu sozialem und wirtschaftlichem Aufstieg, die von der Regierung billigend in Kauf genommen wurden, trugen dazu bei, dass sich der Vermögensunterschied in Amerika vergrößerte. Die Folgen der Diskriminierung zeigen sich heute beispielsweise in der niedrigen Erwerbsquote einiger gesellschaftlicher Gruppen und hohen Schuldenbergen. Mit letzterem haben vor allem Millennials zu kämpfen. Aufgrund hoher Studiengebühren sind sie am Ende ihrer Ausbildung mit einem Schuldenberg konfrontiert, den sie über Jahrzehnte abtragen müssen und der ihre wirtschaftliche Absicherung in der Zukunft gefährdet. Diese Verschuldung beeinträchtigt ihre Möglichkeiten, eigenen Wohlstand aufzubauen und diesen in die amerikanische Wirtschaft einzubringen. Warren macht hierfür Politik und Reiche gleichermaßen verantwortlich: „For decades, we’ve allowed the wealthy to pay less while burying tens of millions of working Americans in education debt“ (Warren 2019f). Durch benachteiligende Gesetze, die die Interessen der Reichen priorisieren und auf Konzerne konzentriert waren, klafft die Schere zwischen Arm und Reich in den USA immer weiter auf.
- **Problembewertung basierend auf moralischen oder anderen Werten**
  *The wealthy and well-connected, corporations* und *the government* werden innerhalb des *wealth*-Frames als Ursache der *wealth gap* in einem negativen Licht präsentiert. Es wird als zutiefst unmoralisch dargestellt, sich in einem Land, in dem der *American Dream* zentraler Teil des Wertesystems ist, auf Kosten hart arbeitender Amerikaner:innen zu bereichern. Dieses Empfinden nutzt Warren, um eine Bewertung der Situation zu kommunizieren. Sie beschuldigt die Vermögenden, sich auf Kosten der Mittelschicht bereichert zu haben: „a small group of families has taken a massive amount of the wealth American workers have produced, while America’s middle class has been hollowed out“ (2019h). Auch bezüglich *corporations* schlägt sie im Rahmen des Frames eine Bewertung vor: „I saw the consequences of the coal industry’s abandonment of the communities that made their shareholders and their executives wealthy – stolen pensions, poisoned miners, and ruined land and water“ (2019g). Dem Publikum wird damit eine Bewertung von Unternehmen präsentiert, in der sich diese ohne Rücksicht auf Verluste bereichern. Die dritte unmoralische Instanz ist *the government*. Politiker:innen in Washington „care more about protecting the wealthy from paying their fair share than they do about solving (…) urgent national problems“ (Warren 2019i). Dieses Framing zielt auch auf die Probleme der Millennials ab. Sie kämpfen darum, eine wirtschaftlich sichere Existenz in einer unsicheren Arbeitsmarktsituation aufzubauen und haben das Gefühl, von der Regierung um ihre Möglichkeiten und ihre politische Einflussnahme gebracht worden zu sein. Diese Wahrnehmungen bilden eine Basis für die erfolgreiche Resonanz des *wealth-*Frames.
- **Handlungsempfehlung zur Lösung dieser Probleme**
  Zur Lösung des Ungleichheits-Problems schlägt Warren Gesetzesänderungen wie die Vermögenssteuer vor, ihre *Ultra-Millionaire Tax*. Anders als beispielsweise Bernie Sanders bewegt sie sich aber weiterhin im kapitalistischen System und will Besitzverhältnisse nicht grundlegend ändern. Die Top-Vermögenden sollen nicht enteignet, aber stärker belastet werden als in der Vergangenheit. Sie greift damit das veränderte Wertesystem vieler Amerikaner:innen auf, die die extremen Auswüchse des Kapitalismus kritischer sehen als früher. Es gilt: Die Superreichen profitierten von der amerikanischen Infrastruktur und Gesellschaft und haben sich auf Kosten anderer bereichert, also sollen sie nun ihren *fair share* bezahlen; gleichzeitig stehen so mehr finanzielle Mittel zur Förderung von Chancengleichheit zur Verfügung, ohne die Mittelschicht mit Steuern zu belasten. Um die tief verwurzelte Abneigung vieler Amerikaner:innen gegen Steuern zu umgehen wählt Warren ihre Formulierungen jedoch sorgfältig. Sie plant, jährlich nur „two-cent tax on every dollar of wealth above $ 50 million“ (2019d) zu erheben und „asks the wealthiest families in America“, eine „small annual tax“ auf ihr Vermögen zu bezahlen (2019b). Die Abwesenheit vehementer Forderungen ist auffällig. Sie betont stattdessen den Beitrag eines *fair share*. Warren will, dass die *Ultra-Millionaire Tax *die Einnahmen generiert, mit denen alle Amerikaner:innen die „wealth-building opportunities“ (2019d) bekommen, die ihnen bisher verwehrt blieben. Weitere Gesetzesvorhaben zu Arbeitnehmerschutz und *Social Security* sollen ebenfalls helfen, für Chancengleichheit „for all Americans“ zu sorgen. Wenn die Privilegierten also einen kleinen Teil beitragen und dadurch dem Rest der Bevölkerung mehr Chancen offenstehen, kann die Ungleichheit bekämpft werden und somit die gesamte amerikanische Wirtschaft profitieren. Dies zumindest schlägt Warren im *wealth*-Frame als Interpretation der Debatte um ökonomische Ungleichheit und deren mögliche Abhilfe vor.

## (Vorwahl‑)Niederlage trotz überzeugender Narrative: Mögliche Ursachen und Schlussfolgerungen

Donald Trumps Präsidentschaft, das Wahljahr 2020 und nicht zuletzt die weltweite Covid-19-Pandemie haben die Spaltung in den USA weiter vertieft und viele politische Themen an den Rand gedrängt. In diesem Kontext ist leicht zu übersehen, dass in den vergangenen Jahren eine Verschiebung des politischen Diskurses im linken Lager der USA stattgefunden hat und sich die Wählerschaft auch von progressiven Ideen mobilisieren lässt. Vor allem junge Leute wollen alternative Ideen zum politischen Mainstream umgesetzt sehen und unterstützen Kandidat:innen des linken Spektrums. Sie wollen tiefgreifende Veränderungen im politischen System und eine Umkehr der etablierten Politik. Diese Tendenzen konnten sich bei der Kandidatensuche der Demokratischen Partei im Vorwahlkampf 2019/2020 allerdings nicht durchsetzen.

Dieses Papier untersuchte die Kommunikationsstrategie Elizabeth Warrens im Präsidentschaftswahlkampf 2019/2020, deren Aussichten auf Erfolg zu Beginn vielversprechend waren. Sie konnte zu vielen Themen Schwerpunkte setzen und war laut New York Times eine Zeit lang der „national pacesetter“ (Goldmacher und Herndon 2020). So trieb sie oftmals den Diskurs und die Agenda des Wahlkampfes voran. Die Analyse der von Warren genutzten strategischen Frames zeigt, dass ihre Kampagne sowohl die Sorgen großer Teile der amerikanischen Bevölkerung als auch soziale Vorhaben in den Vordergrund rücken wollte. Das vielleicht etablierteste Narrativ der US-Gesellschaft, der *American Dream *und dessen Versprechen auf ein besseres Leben, wurde hier genutzt, um ihre politische Agenda nachvollziehbar zu machen und Identifizierungspunkte für das Publikum zu schaffen. Sie betont, wie stark die USA durch die bisherige Politik und die Gier der Reichen fehlgeleitet wurde. Für Probleme wie fehlende Anerkennung harter Arbeit und die dadurch größer werdende Schere zwischen Arm und Reich, welche sich auch auf die Wirtschaft auswirkt, präsentierte die Warren-Kampagne Schuldige und mögliche Lösungen. Sie bot verschiedene Bewertungen wie das *rigged *oder *unfair system* an und stellte zudem Unternehmen und die Regierung als ausbeuterisch und paktierend dar, die der hart arbeitenden Bevölkerung faire Chancen verwehren. Als eine Lösung wurde die *Ultra-Millionaire Tax* diskutiert, die die Wohlstandsverteilung ausbalancieren soll. Diese Kommunikationsstrategie Warrens schien verstärkt auf eine jüngere Generation ausgerichtet, die zunehmend progressive Vorhaben unterstützt.

Der Erfolg dieser Strategie muss für den Präsidentschaftswahlkampf 2019/2020 jedoch als gescheitert betrachtet werden. Ohne einen Anspruch auf Vollständigkeit zu erheben, sollen im Folgenden einige mögliche Gründe für Warrens Misserfolg genannt werden. Warren versuchte von Anfang an, sich als (weibliche) Alternative zu dem moderaten Biden und dem progressiven Sanders zu platzieren. Die Wählerschaft scheint sich allerdings lieber für die eine oder andere Seite entschieden zu haben, weniger für einen Kompromiss in Form von Warren (Friedersdorf 2020). Auch der Eindruck von Authentizität, den Warren zu vermitteln versuchte, bekam schon vor den Vorwahlen Risse. Beispielsweise blieben ihre Aussagen zu *Medicare for All* (einem universellen Krankenversicherungssystem, finanziert durch den Staat), *dem* zentralen Schwerpunkt der Linken, oft widersprüchlich, was ihr von linken und moderaten Mitbewerber:innen als nicht authentisch und ausweichend ausgelegt wurde (Friedersdorf 2020). Zudem revidierte sie ihre Meinung zu weiteren zentralen progressiven Unumstößlichkeiten, wie etwa die Finanzierung der Wahlkampagne durch Super-PACs, und akzeptierte deren Spenden, statt ihren Wahlkampf ausschließlich durch individuelle Kleinspender:innen zu finanzieren, wie es die Sanders-Kampagne tat. Sicherlich war auch das eisig gewordene Verhältnis zwischen den Kampagnen von Warren und Sanders problematisch. Die öffentliche Aussage durch das Warren-Team (Herndon und Martin 2020), Sanders traue einer weiblichen Kandidatin die Präsidentschaftswahl nicht zu, scheint die letzten Verbindungen zwischen Warren und Sanders zerschnitten zu haben. Obwohl der Einfluss von Sexismus bei politischen Wahlen nicht außer Acht gelassen werden kann, schien dieser Vorwurf gerade gegenüber Sanders vielen nicht glaubwürdig und scheint die Skepsis der Wählerschaft Warren gegenüber verstärkt zu haben.

Keiner dieser Faktoren – die Positionierung als Kompromiss zwischen Biden und Sanders, ihr Schwanken bezüglich progressiven Schwerpunktthemen und die daraus resultierende schwindende Authentizität, sowie das Überwerfen mit der Sanders-Kampagne – war wohl als einziger entscheidend für Warrens Scheitern im Vorwahlkampf, gemeinsam trugen sie aber sicherlich zu ihrer sinkenden Unterstützung bei. Zudem konnte Warren, anders als Sanders, bisher keine entscheidenden Mehrheiten außerhalb des weißen akademischen Wählerfeldes erreichen (Goldmacher und Herndon 2020). Obwohl ihre Reformideen vor allem auf *working class* Wählergruppen ausgerichtet sind, blieb sie vor allem die Kandidatin des akademischen Bildungsbürgertums. Hierfür machen manche Beobachter:innen auch Äußerungen im Wahlkampf verantwortlich, die wenig authentisch und eher nach „woke virtue-signaling“ (Friedersdorf 2020) klangen. Dies bringt zum Ausdruck, dass eine Sprache, die sich teilweise an den identitätspolitischen Debatten des linken akademischen Umfelds orientiert, Wählergruppen außerhalb dieses Spektrums abschreckt (Friedersdorf 2020). Auch bei *swing voters*, die noch unentschlossen waren, Trump aber als Präsidenten ablehnten, konnte Warren augenscheinlich nicht punkten. Hier war oftmals nicht *structural change* gefragt, sondern eine Rückkehr zu einer gewissen Normalität nach der Trump-Präsidentschaft, inklusive eines konstanten wirtschaftlichen Wachstums. Dies schienen die meisten eher Joe Biden als Präsident zuzutrauen, als einer Kandidatin Elizabeth Warren.

Obwohl Warren im Wahlkampf 2020 scheiterte, ist nicht zu vernachlässigen, dass sie weiterhin Einfluss auf den linken Diskurs in und um die Demokratische Partei hat. Auch, wenn sie nicht Präsidentschaftskandidatin wurde, ist sie als Senatorin immer noch eine entscheidende Figur des progressiven Flügels der Demokratischen Partei, die auch auf die neue Biden-Regierung Einfluss ausübt, etwa bei Personalentscheidungen (Rappeport 2021). An ihrem Beispiel verdeutlicht dieses Papier, wie progressive Reformvorschläge konkret im politischen Diskurs kommuniziert werden. Wie Warren aber zeigt, bedeuten eine professionell geführte Kampagne und ein detaillierter Reformplan nicht automatischen Erfolg als Kandidatin. Eine genauere Analyse der Gründe, die zu Warrens Scheitern im Wahlkampf geführt haben, könnte sicherlich noch genauere Erkenntnisse liefern.

Die Wahl eines moderaten Kandidaten zum Präsidenten zeigt, dass sich der progressive Flügel in der Demokratischen Partei nicht durchsetzen konnte. Dennoch ist das Erstarken dieses Flügels und das Herausbilden progressiver politischer Schwergewichte wie Alexandria Ocasio-Cortez, Bernie Sanders oder Elizabeth Warren auf nationaler Ebene nicht von der Hand zu weisen. Allein die Tatsache, dass progressive Politiker:innen wie Sanders und Warren lange erfolgreich im Präsidentschaftswahlkampf waren, wäre vor einigen Jahrzehnten noch undenkbar gewesen. Auch die Tatsache, dass Biden mit der progressivsten Agenda antrat, die ein Präsidentschaftskandidat einer der großen Parteien jemals hatte (Beinhart 2020; Warren 2020b), weist darauf hin, dass sich zumindest ein Teil des politischen Diskurses in den USA nach links zu verschieben scheint und dass vor allem die linke Basis tiefgreifende Veränderungen im System der USA durchsetzen will. Zwar sind einige dieser Vorschläge, wie etwa eine universelle Krankenversicherung, aus europäischer Perspektive nicht bahnbrechend, sondern schon lange Teil sozialdemokratischer Politik, doch in den USA war eine solche Politik lange nicht Teil des politischen Mainstreams. Nach dem Einzug des neuen Präsidenten ins Weiße Haus und einem Mehrheitsgewinn der Demokraten in beiden Kammern des Kongresses wird es von progressiver Seite nun auch Forderungen nach einer konkreten Umsetzung progressiver Ideen geben.

## Footnotes

1 Zu finden auf ihrer Website https://elizabethwarren.com/plans, Stand Oktober 2019.

2 Politiker:innen der Demokratischen Partei setzten verstärkt auf die Kommunikation progressiver Inhalte, um Wähler:innen anzusprechen. Beispielsweise fanden sich zahlreiche Bewerber:innen um die Präsidentschaftskandidatur unter den zehn am progressivsten abstimmenden Senator:innen des Landes. Vorne dabei waren Elizabeth Warren mit 98,95 %, sie ist damit auf Platz 2 der progressivsten *voting records*, Kamala Harris auf Platz 3 mit 97,71 % und Cory Booker mit 97,46 % auf Platz 7 (ProgressivePunch 2019). Ein Blick auf die Umfragewerte der Kandidat:innen vor dem Super Tuesday zeigt außerdem, dass linke Kandidat:innen wie Elizabeth Warren (24 %), Bernie Sanders (15 %) oder Julián Castro (<1 %) auf etwa 40 % Zustimmung kamen und zu dritt fast genauso viel Unterstützung hatten wie die als moderat geltenden Kandidat:innen, die auf etwa 47 % kamen (Lee et al. 2019; The Economist 2019). Obwohl Joe Biden, der die Vorwahlen gewann, als moderat gilt, trat er bei der Präsidentschaftswahl mit einer Agenda an, die in mancherlei Hinsicht als progressiv eingeschätzt worden ist (Milbank 2020; JoeBidenPresidentialCampaign 2020). Linke Politik scheint also einen größeren Einfluss zu haben. Dies zeigt sich auch darin, dass inzwischen sogar Republikaner:innen Themen wie den Erlass von Studienschulden ansprechen, wie zum Beispiel A. Wayne Johnson, der das *student-loan *System als „fundamentally broken“ bezeichnete (Mitchell 2019).

3 Die Sanders Kampagne betont stehts den ‚grassroots‘ Charakter der Wahlkampagne, z. B. mit den Slogans „This is *your *movement!“ oder „Not Me – Us!“ (BernieSandersCampaign 2021).

4 Zu dieser Generation gehören alle, die in etwa zwischen 1981 und 1996 geboren wurden.

5 Beispielsweise setzt *Instagram* als eine der wachsenden Plattformen auf Bilder, *Stories *und *Reels*. Zusätzlich gibt es Instagram TV (IGTV), über das auch längere Videos geteilt werden können.

6 Warrens Vorschlag sieht vor, eine zweiprozentige Steuer auf Vermögen über 50 Mio. Dollar zu erheben, was ungefähr die 70.000 reichsten Familien der USA betreffen würde. Bei einem Vermögen von einer Milliarde Dollar oder mehr würde zusätzlich ein Prozent Steuer erhoben. Wie viele Einnahmen wirklich generiert werden könnten und ob diese für weitreichende Pläne ausreichen würden, bleibt umstritten (Schwartz und Gates 2019).

7 Die Zahlen sind jeweils Stand Oktober 2019, während des Vorwahlkampfs, und Stand Januar 2021. Im Vergleich: Joe Biden (1,5 Mio. FB – 8,5 Mio., 3,8 Mio. – 24,5 Mio. Twitter, 1,3 Mio. – 15,6 Mio. Instagram); Kamala Harris (1,1 Mio. – 4 Mio. FB, 3,2 Mio. – 14,9 Mio. Twitter, 2 Mio. – 14,8 Mio. Instagram); Pete Buttigieg (465 – 713 Tsd. FB, 1,4 Mio. – 3,1 Mio. Twitter, 923 Tsd. – 1,3 Mio. Instagram).

8 Vgl. https://www.facebook.com/ElizabethWarren/.

9 Vgl. https://twitter.com/ewarren?lang=de.

10 Vgl. https://www.instagram.com/elizabethwarren/?hl=de.

11 https://2020.elizabethwarren.com/.

12 Die Zahl hat sich bis zum 27.01.2021 auf 81 erhöht. Neu dazugekommen ist beispielsweise der Plan: „Restoring Integrity and Competence to Government After Trump“.

13 Framing als Konzept wird in zahlreichen Forschungsbereichen und auf unterschiedliche Untersuchungsgegenstände angewendet. Deshalb existiert in der Frame-Forschung eine Vielzahl von Ansätzen. Ein verbreiteter Ansatz ist der des Linguisten George Lakoff (Lakoff 2014). Dieser versteht Framing als einen kognitiven Prozess, eine unbewusste menschliche Tendenz dazu, die Welt mit Hilfe existierender Deutungsmuster, die auf Vorwissen und Erfahrungen beruhen, zu verstehen. Dieses unbewusste Rezeptionsverhalten können sich politische Akteure und Akteurinnen wiederum zunutze machen und Frames strategisch einsetzen (siehe auch Wehling 2016). Ansätze aus der Politik- und Kommunikationsforschung betrachten Framing daher häufig als Strategie, mit deren Hilfe Themen in einem Diskurs gezielt und in einer bestimmten Weise platziert und Interpretationen vorgegeben werden (Gamson und Modigliani 1989; Entman 1993; Matthes 2012, 2014). Die Analyse des vorliegenden Artikels folgt einem Verständnis von Framing als bewusst eingesetzter Kommunikationsstrategie.

14 Der Korpus umfasst alle Pläne, die Stand 18. Oktober 2019 auf der Website Warrens vorgestellt wurden.

15 Zur Analyse des Korpus wurde die Software AntConc eingesetzt.

16 In der Linguistik bezeichnet man mit Kollokationen Wörter, die in einem Text oder Korpus häufig benachbart auftreten (Brezina 2018, S. 67). Kollokationen weisen also auf die Nutzung präferierter Kombinationen von Wörtern hin. Diese fällt besonders auf, wenn Wortkombinationen unerwartet sind. Gängige Kollokationen lassen sich empirisch in Texten und Korpora nachweisen.

17 Um die benachbarte Nutzung von Wörtern zu messen, wurde der MI Score (Mutual Information Score) herangezogen. Dieser gibt an, wie exklusiv die Kollokation von Begriffen ist. Je höher der MI Score, desto häufiger werden zwei Begriffe ausschließlich benachbart verwendet, selbst wenn die Kollokation im Korpus nur ein- oder zweimal auftritt (Brezina 2018, S. 70–71). Für die Kollokations-Analyse wurden folgende Parameter festgelegt: Der MI Score sollte ≥7 betragen und die minimale Frequenz der Kollokation zwei. Betrachtet wurden Ergebnisse, die sich innerhalb einer Spanne von bis zu vier Positionen jeweils links und rechts des gesuchten Begriffs befanden.

## References

[CR1] Ajilore O (2019). The United States is not ready for a recession, but it can be.

[CR74] AntConc (Version 3.5.8) [Computer Software]. Waseda University. Available from https://www.laurenceanthony.net/software. Zugegriffen: May 10, 2021.

[CR3] Beinhart, Peter. 2020. Biden goes big without sounding like it. https://www.theatlantic.com/ideas/archive/2020/08/joe-bidens-big-bold-and-very-quiet-agenda/614878/. Zugegriffen: 21. Jan. 2021.

[CR4] Benford RD, Snow DA (2000). Framing processes and social movements: an overview and assessment. Annual Review of Sociology.

[CR5] Bernie Sanders Campaign. 2021. Bernie Sanders Website. https://berniesanders.com/. Zugegriffen: 2. Mai 2021.

[CR6] Brezina V (2018). Statistics in corpus linguistics. A practical guide.

[CR7] Chetty R, Grusky D, Hell M, Hendren N, Manduca R, Narang J (2016). The fading American dream: trends in absolute income mobility since 1940.

[CR8] DeCosta-Klipa, Nik. 2018. Read the transcript of Elizabeth Warren’s re-election victory speech. https://www.boston.com/news/politics/2018/11/07/elizabeth-warren-re-election-victory-speech. Zugegriffen: 7. Okt. 2019.

[CR9] Druckman JN, Lupia A (2016). Preference change in competitive political environments. Annual Review of Political Science.

[CR10] Economist. 2019. Warrensworld: Elizabeth Warren’s many plans would reshape American capitalism. *The Economist*.

[CR11] Entman RM (1993). Framing: toward clarification of a fractured paradigm. Journal of Communication.

[CR12] Friedersdorf, Conor. 2020. Why Elizabeth Warren Lost. https://www.theatlantic.com/ideas/archive/2020/03/why-elizabeth-warren-lost/608029/. Zugegriffen: 8. Febr. 2021.

[CR13] Gadinger F, Jarzebski S, Yildiz T (2014). Politische Narrative: Konzepte – Analysen – Forschungspraxis.

[CR14] Gamson WA, Modigliani A (1989). Media discourse and public opinion on nuclear power: a constructionist approach. American Journal of Sociology.

[CR15] Gerhards J, Rucht D (1992). Mesomobilization: organizing and framing in two protest campaigns in west Germany. American Journal of Sociology.

[CR16] Goldmacher, Shane, und Astead W. Herndon. 2020. Elizabeth Warren, once a front-runner, drops out of presidential race. https://www.nytimes.com/2020/03/05/us/politics/elizabeth-warren-drops-out.html. Zugegriffen: 13. Jan. 2021.

[CR17] Hacker JS, Pierson P (2010). Winner-take-all politics: public policy, political organization, and the precipitous rise of top incomes in the United States. Politics & Society.

[CR18] Hauke N, Gadinger F, Jarzebski S, Yildiz T (2014). Die grüne Revolution an der Tankstelle? Die Relevanz politischer Narrative am Beispiel der Einführung des Biokraftstoffes E10. Politische Narrative.

[CR19] Herndon, Astead W., und Jonathan Martin. 2020. Warren says sanders told her a woman could not win the presidency. https://www.nytimes.com/2020/01/13/us/politics/bernie-sanders-elizabeth-warren-woman-president.html. Zugegriffen: 10. Mai 2021.

[CR20] Jacobs, Samuel P. 2011. Elizabeth Warren: I created occupy Wall Street. https://www.thedailybeast.com/elizabeth-warren-i-created-occupy-wall-street. Zugegriffen: 7. Okt. 2019.

[CR21] Joe Biden Presidential Campaign. 2020. President Barack Obama endorses Joe Biden for President: YouTube. https://www.youtube.com/watch?v=5-s3ANu4eMs. Zugegriffen: 20. Juli 2021.

[CR22] Kearney MS, Hershbein B, Boddy D (2015). The future of work in the age of the machine. Hamilton project framing paper: the Hamilton project, Brookings.

[CR23] Krugman P, Boushey H, DeLong JB, Steinbaum M (2017). Why we’re in a new gilded age. After Piketty: the agenda for economics and inequality.

[CR24] Lakoff G (2014). The all new don’t think of an elephant!: know your values and frame the debate.

[CR25] Lammert C, Vormann B, Oswald M (2019). When Inequalitities matter most: the crisis of democracy as a crisis of trust. Mobilization, representation, and responsiveness in the American democracy.

[CR26] Landau MJ, Keefer LA, Rothschild ZK (2014). Epistemic motives moderate the effect of metaphoric framing on attitudes. Journal of Experimental Social Psychology.

[CR27] Lee, Jasmine C., Annie Daniel, Rebecca Lieberman, Blacki Migliozzi, und Alexander Burns. 2019. Which democrats are leading the 2020 presidential race? https://www.nytimes.com/interactive/2020/us/elections/democratic-polls.html. Zugegriffen: 24. Okt. 2019.

[CR28] Matthes J (2012). Framing politics: an integrative approach. American Behavioral Scientist.

[CR29] Matthes J (2014). Framing. Konzepte.

[CR30] Milbank, Dana. 2020. Opinion: Bidens Temperament is moderate, his agenda is transformative. https://www.washingtonpost.com/opinions/2020/10/27/bidens-temperament-is-moderate-his-agenda-is-transformative/. Zugegriffen: 13. Jan. 2021.

[CR31] Milkman R (2017). A new political generation: millennials and the post-2008 wave of protest. American Sociological Review.

[CR32] Mitchell, Josh. 2019. Trump Education Official to Resign and Call for Mass Student-Loan Forgiveness. *The Wall Street Journal*. 24.10.2019.

[CR33] Naples N (2003). Feminism and method: ethnography, discourse analysis, and activist research.

[CR34] Nepstad SE, Smith J, Johnston H (2002). Creating transnational solidarity: The use of narrative in the U.S.-central America peace movement. Globalization and resistance: transnational dimensions of social movements.

[CR35] Kleis R, Nielsen (2013). Mundane internet tools, the risk of exclusion, and reflexive movements—occupy wall street and political uses of digital networked technologies. The Sociological Quarterly.

[CR36] NYT. 2021. Businesses are closing, and job losses are mounting. https://www.nytimes.com/live/2020/12/17/business/us-economy-coronavirus#new-unemployment-claims-remain-far-above-historical-levels. Zugegriffen: 13. Jan. 2021.

[CR37] Olsen KA (2014). Telling our stories: narrative and framing in the movement for same-sex marriage. Social Movement Studies.

[CR38] Oswald M (2019). Strategisches Framing : Eine Einführung.

[CR39] Pew-Research-Center. 2017. How wealth inequality has changed in the U.S. since the Great Recession, by race, ethnicity and income. https://www.pewresearch.org/fact-tank/2017/11/01/how-wealth-inequality-has-changed-in-the-u-s-since-the-great-recession-by-race-ethnicity-and-income/. Zugegriffen: 10. Mai 2021.

[CR40] Pew-Research-Center. 2019. Share of U.S. adults using social media, including Facebook, is mostly unchanged since 2018. https://www.pewresearch.org/fact-tank/2019/04/10/share-of-u-s-adults-using-social-media-including-facebook-is-mostly-unchanged-since-2018/. Zugegriffen: 10. Mai 2021.

[CR41] Pew-Research-Center. 2020. Unemployment rose higher in three months of COVID-19 than it did in two years of the Great Recession. https://www.pewresearch.org/fact-tank/2020/06/11/unemployment-rose-higher-in-three-months-of-covid-19-than-it-did-in-two-years-of-the-great-recession/. Zugegriffen: 13. Jan. 2021.

[CR42] Piketty T, Saez E (2014). Inequality in the long run. Science.

[CR43] Polletta F (1998). Contending stories: narrative in social movements. Qualitative Sociology.

[CR44] Polletta F (1998). “It was like a fever ...” narrative and identity in social protest. Social Problems.

[CR45] Progressive Punch. 2019. Progressive score US senators. https://progressivepunch.org/scores.htm?house=senate. Zugegriffen: 29. Okt. 2019.

[CR46] Purdy, Jedediah. 2017. America’s new opposition. *New Republic*. https://newrepublic.com/article/140268/americas-new-opposition-left-resistance-trump. Zugegriffen: 20. Juli 2021.

[CR48] Rappeport, Alan, und Thomas Kaplan. 2019. Democrat’s plans to tax wealth would reshape U.S. economy. https://www.nytimes.com/2019/10/01/us/politics/sanders-warren-wealth-tax.html. Zugegriffen: 7. Okt. 2019.

[CR47] Rappeport, Alan. 2021. A year after ending her presidential bid, Warren wields soft power in Washington. https://www.nytimes.com/2021/03/22/us/politics/elizabeth-warren-taxes-infrastructure.html. Zugegriffen: 2. Mai 2021.

[CR49] Rauscher N, Oswald M, Johann M (2018). Von Occupy Wall Street zu den ‚nasty women‘ – Digitale Kommunikation als Partizipationsmöglichkeit neuer Protestströmungen. Strategische Politische Kommunikation im digitalen Wandel: Interdisziplinäre Perspektiven auf ein dynamisches Forschungsfeld.

[CR50] Russman U, Svensson J (2016). Studying organizations on Instagram. Information.

[CR51] Sabatier PA (1998). The advocacy coalition framework: revisions and relevance for Europe. Journal of European Public Policy.

[CR52] Schwartz, Nelson D., und Guilbert Gates. 2019. Democrats want to tax the rich. Here’s how those plans would work (or not). https://www.nytimes.com/interactive/2019/09/24/business/economy/wealth-tax-rich.html. Zugegriffen: 24. Okt. 2019.

[CR53] Shiller RJ (2017). Narrative economics. The American Economic Review.

[CR54] Shiller RJ (2018). Narrative economics: how stories go viral & drive major economic events.

[CR55] Taylor, Kate. 2019. Elizabeth Warren formally announces 2020 presidential bid in Lawrence, Mass. https://www.nytimes.com/2019/02/09/us/politics/elizabeth-warren-2020.html. Zugegriffen: 7. Okt. 2019.

[CR56] The Economist. 2019. Who is ahead in the Democratic primary race? https://projects.economist.com/democratic-primaries-2020/. Zugegriffen: 29. Okt. 2019.

[CR57] Thunert, Martin. 2021. Wahlausgang mit gegenläufigen Botschaften. https://regierungsforschung.de/wahlausgang-mit-gegenlaeufigen-botschaften/. Zugegriffen: 13. Jan. 2021.

[CR58] Walker ZS (2014). Populism and myth in the rhetoric of Elizabeth Warren.

[CR59] Warren, Elizabeth. 2012a. Elizabeth Warren’s full DNC speech. https://www.youtube.com/watch?v=FzspAfNkGz0. Zugegriffen: 7. Okt. 2019.

[CR60] Warren, Elizabeth. 2012b. Elizabeth Warren victory speech. https://www.c-span.org/video/?309220-1/elizabeth-warren-victory-speech. Zugegriffen: 7. Okt. 2019.

[CR61] Warren, Elizabeth. 2019a. Affordable higher education for all. What Elizabeth Will do. https://elizabethwarren.com/plans/affordable-higher-education. Zugegriffen: 26. Okt. 2019.

[CR62] Warren, Elizabeth. 2019b. Defend & create American jobs. What Elizabeth Will do. https://elizabethwarren.com/plans/american-jobs. Zugegriffen: 19. Febr. 2019.

[CR63] Warren, Elizabeth. 2019c. Empowering American workers and raising wages. What Elizabeth Will do. https://elizabethwarren.com/plans/empowering-american-workers. Zugegriffen: 26. Okt. 2019.

[CR64] Warren, Elizabeth. 2019d. Ending the opioid crisis. What Elizabeth Will do. https://elizabethwarren.com/plans/end-opioid-crisis. Zugegriffen: 26. Okt. 2019.

[CR65] Warren, Elizabeth. 2019e. Expanding social security. What Elizabeth Will do. https://elizabethwarren.com/plans/social-security. Zugegriffen: 26. Okt. 2019.

[CR66] Warren, Elizabeth. 2019f. Leveling the playing field for entrepreneurs of color. What Elizabeth Will do. https://elizabethwarren.com/plans/leveling-field-entrepreneurs-color. Zugegriffen: 26. Okt. 2019.

[CR67] Warren, Elizabeth. 2019g. A new farm economy. What Elizabeth Will do. https://elizabethwarren.com/plans/new-farm-economy. Zugegriffen: 26. Okt. 2019.

[CR68] Warren, Elizabeth. 2019h. Promoting Competitive Markets. What Elizabeth Will Do. https://elizabethwarren.com/plans/promoting-competitive-markets. Zugegriffen: 26. Okt. 2019.

[CR69] Warren, Elizabeth. 2019i. Universal child care. What Elizabeth Will do. https://elizabethwarren.com/plans/universal-child-care. Zugegriffen: 19. Febr. 2019.

[CR70] Warren, Elizabeth. 2020a. Elizabeth Warren speech transcript on dropping out of the presidential race. https://www.rev.com/blog/transcripts/elizabeth-warren-speech-transcript-on-dropping-out-of-presidential-race. Zugegriffen: 13. Jan. 2021.

[CR71] Warren, Elizabeth. 2020b. New York times events, dealbook: senator Elizabeth Warren discusses the post-election outlook. https://www.youtube.com/watch?v=AH7Xb-oUUrs&ab_channel=NewYorkTimesEvents. Zugegriffen: 21. Jan. 2021.

[CR72] Wehling E (2016). Politisches Framing: wie eine Nation sich ihr Denken einredet – und daraus Politik macht. Edition Medienpraxis.

[CR73] Yildiz T, Gadinger F, Smith C (2018). Narrative Legitimierung: exekutive, repräsentative und subversive Erzählstrategien in der Überwachungskontroverse. Leviathan.

